# Retinal Dysfunction in Alzheimer’s Disease and Implications for Biomarkers

**DOI:** 10.3390/biom11081215

**Published:** 2021-08-16

**Authors:** Chunyan Liao, Jinying Xu, Yu Chen, Nancy Y. Ip

**Affiliations:** 1 Chinese Academy of Sciences Key Laboratory of Brain Connectome and Manipulation, Shenzhen Key Laboratory of Translational Research for Brain Diseases, The Brain Cognition and Brain Disease Institute, Shenzhen Institute of Advanced Technology, Chinese Academy of Sciences, Shenzhen-Hong Kong Institute of Brain Science—Shenzhen Fundamental Research Institutions, Shenzhen 518055, China; cy.liao@siat.ac.cn (C.L.); jy.xu@siat.ac.cn (J.X.); 2 Guangdong Provincial Key Laboratory of Brain Science, Disease and Drug Development, Shenzhen-Hong Kong Institute of Brain Science, HKUST Shenzhen Research Institute, Shenzhen 518057, China; 3 Shenzhen College of Advanced Technology, University of Chinese Academy of Sciences, Beijing 100049, China; 4 Division of Life Science, Molecular Neuroscience Center, and State Key Laboratory of Molecular Neuroscience, The Hong Kong University of Science and Technology, Hong Kong 999077, China

**Keywords:** Alzheimer’s disease, retinal abnormality, biomarker, amyloid-beta, tau, vascular changes

## Abstract

Alzheimer’s disease (AD) is a progressive neurodegenerative disorder that manifests as cognitive deficits and memory decline, especially in old age. Several biomarkers have been developed to monitor AD progression. Given that the retina and brain share some similarities including features related to anatomical composition and neurological functions, the retina is closely associated with the progression of AD. Herein, we review the evidence of retinal dysfunction in AD, particularly at the early stage, together with the underlying molecular mechanisms. Furthermore, we compared the retinal pathologies of AD and other ophthalmological diseases and summarized potential retinal biomarkers measurable by existing technologies for detecting AD, providing insights for the future development of diagnostic tools.

## 1. Introduction

Alzheimer’s disease (AD), the most prevalent form of dementia, is a progressive neurodegenerative disorder that features a decline in memory and defects in cognitive functions. The pathological hallmarks of AD include the deposition of extracellular amyloid-beta (Aβ) plaques and the formation of neurofibrillary tangles by the intracellular hyperphosphorylated tau protein [1]. Over the past few decades, there has been significant progress in understanding AD pathophysiology and in the development of intervention strategies. Nevertheless, there is still no effective treatment or adequate diagnostic tools for AD. Pathological changes caused by AD can emerge several years or even decades before neurodegeneration and cognitive impairment occur, making it extremely challenging to implement effective interventions once these clinical symptoms become severe [2]. Therefore, biomarkers for AD are urgently required for the early diagnosis and risk prediction of the disease.

Notably, the presence of retinal aberrations in AD, including Aβ deposition, neurofibrillary tangle aggregation, abnormal retinal microvascular circulation, and thinning of the retinal nerve fiber layer (RNFL), suggests the intriguing possibility of developing easily measurable retinal biomarkers for early-stage AD diagnosis [3]. Herein, we review the current knowledge of retinal dysfunction in AD and the development of potential biomarkers based on such retinal abnormalities. We also discuss the pathophysiology and related biomarkers for AD that overlap with other ophthalmological diseases.

## 2. Retinal Changes in Alzheimer’s Disease and Implications for Biomarkers

As the incidence of AD increases worldwide, largely due to global population aging, enormous efforts have been made to develop effective approaches for screening high-risk individuals before AD symptoms occur or identifying patients with AD at early stages. For example, various methods have been used to detect neurotoxic Aβ_42_ and Aβ_40_, phosphorylated tau, total tau, and neurofilament light chain in cerebrospinal fluid (CSF) and plasma; changes in the absolute quantities or ratios of these biomarkers are strongly associated with AD progression [4,5]. In addition, positron emission tomography (PET) is used to visualize Aβ and tau deposition or measure the metabolic rate in the brains of patients, while magnetic resonance imaging (MRI) is used to detect brain atrophy [6,7]. However, these methods have limited utility for population-level screening owing to high cost, invasiveness, or ineffectiveness in discerning changes in early-stage AD or identifying high-risk individuals.

The eyes are the primary visual sensory organs. The retina comprises the neural retina and retinal pigment epithelium (RPE) and is a crucial component of the central nervous system [8]. The neural retina develops from the neural tube and is enriched with neurons including retinal ganglion cells (RGCs), photoreceptor cells, bipolar cells, amacrine cells, and horizontal cells [8,9]. In addition, three main types of glia cells support retinal homeostasis: Müller glial cells, astrocytes, and microglia [10]. The neural retina comprises three cellular layers including the ganglion cell layer (GCL), inner nuclear layer (INL), and outer nuclear layer (ONL). The ONL is covered with rod and cone photoreceptors, which transmit visual information to RGCs located in the GCL via interneurons within the INL. The neurons of the INL, which include bipolar, horizontal, and amacrine cells, process and transduce signals to the ganglion cells. Meanwhile, the synaptic junctions in the retina congregate in the outer plexiform layer (OPL) and inner plexiform layer (IPL). The OPL covers the synapses between the photoreceptors and INL cells including horizontal and bipolar cells. The IPL is occupied by synapses between the INL cells and RGCs. Thus, visual signals are transmitted from the RGCs to the brain via the optic nerve [11].

Therefore, the retina is considered an extension of the brain as well as a potential source of AD biomarkers that can be evaluated noninvasively [12,13]. Accordingly, retinal biomarkers have been identified for the early screening of individuals at high risk of AD [14,15]. Nevertheless, the mechanisms underlying the RGC and the optic nerve degeneration involved in visual abnormalities in AD are not well understood. Aβ deposition in the retina is neurotoxic and potentially fatal to RGCs, resulting in RNFL thinning and optic nerve degeneration. Meanwhile, retinal cell functioning can be disrupted by defects in neurotransmitters such as acetylcholine [16,17,18].

### 2.1. Structural Degeneration of the Retina in Early-Stage Alzheimer’s Disease

Structural abnormalities of the retina in AD include optic nerve degeneration, neuronal loss, and reduced thickness of the RNFL and other retinal layers. In 1986, Hinton et al. found that patients with AD showed broad axonal degeneration in the optic nerve, which was verified by *p*-phenylenediamine staining [19]. They subsequently reported RGC degeneration in patients with AD, which featured cell swelling, nuclear disintegration, and a vacuolated cytoplasm [20]. A meta-analysis of optical coherence tomography (OCT) data found that the RNFL was significantly thinner in patients with mild cognitive impairment (MCI) or AD than in healthy people [21]; the loss of RGCs and optic nerve axons may be accounted for by the RNFL thinning [22,23]. Other studies have confirmed this finding [24,25,26], further indicating that such anomalous retinal alterations might appear in early-stage AD.

Blanks et al. quantified the neurons in different retinal regions in AD cases and controls, and found that the former had fewer GCL neurons in the central retina (i.e., 3-mm diameter region) [27]. In particular, the GCL neurons in the fovea (0–0.5-mm eccentricity) exhibited the largest reduction in AD cases compared to that in controls; there were also smaller reductions in the numbers of neurons with the eccentricity of 0.5–1- and 1–1.5-mm in AD cases [27]. Blanks et al. subsequently found that neuronal losses were most dramatic in the superior and inferior quadrants in AD cases compared to those in healthy controls [28].

The macula, approximately 6 mm in diameter, contains the highest concentration of RGCs, whose degeneration is implicated in AD. Neuronal loss in the macular ganglion cell–inner plexiform layer (GC-IPL) is closely associated with patients with MCI or AD, but not cognitively normal controls; macular GC-IPL neuronal loss is associated with MCI more strongly than axonal loss in the RNFL [29]. Nevertheless, another study reported a significant reduction in GCL thickness in all retinal quadrants (i.e., superior, inferior, temporal, and nasal) in patients with AD or MCI compared to age-matched healthy controls; however, the IPL was remarkably reduced in all retinal quadrants in AD, but not in MCI [30]. Meanwhile, the macular ganglion cell complex (GCC), which contains the three innermost layers of the retina (i.e., the RNFL, GCL, and IPL), was significantly reduced in patients with AD compared to healthy controls, although no significant difference in outer retinal layer thickness was observed between groups [31,32].

However, several studies observed no differences in retina parameters between patients with AD and healthy controls, which is contradictory to the abovementioned data. One study showed that the Aβ positive and Aβ negative groups had no statistically significant differences in terms of macular retinal layer thickness and peripapillary RNFL thickness [33]. This apparent discrepancy may arise from the different diagnostic criteria used in these studies or the different disease stages of the AD participants [33]. On the other hand, whether subjects with ophthalmological diseases such as age-related macular degeneration (AMD) and glaucoma are excluded will also affect the statistical results of AD-associated retinal changes [33]. Another study also found no significant differences in RNFL and GCL thickness between patients with AD and controls, owing to the lack of amyloid imaging for accurate diagnosis and limited cohort size [34]. Improvement of diagnosis consistency across cohorts and the use of more specific biomarkers may help solve the discrepancy.

Structural changes of the retina can be detected by comprehensive imaging techniques. For example, various ophthalmological disorders are clinically assessed and diagnosed using spectral-domain OCT, an improved noninvasive method providing two-dimensional, cross-sectional pictures of the morphological structures of the retina at high resolution, as well as three-dimensional volumetric parameters such as RNFL thickness, macular thickness, and macular volume [35,36,37]. This technological development has enabled the observation of the optic nerve topography, RNFL thickness, and macular volume, which are potential clinical biomarkers for early-stage AD diagnosis [13,31,38,39]. Given that the macula includes approximately half of all RGCs and the GC-IPL represents the distribution of RGC bodies and dendrites [40], the finding that macular GC-IPL thickness rather than RNFL thickness is more closely associated with MCI indicates that RGC neuronal loss might start in the macular region [29]. Therefore, macular GC-IPL thinning might be a sensitive indicator of the early changes that occur in the retina in AD [29,41]. However, OCT imaging lacks the specificity to distinguish AD from other degenerative disorders that share retinal layer thinning (e.g., AMD and glaucoma) or optic nerve impairment (e.g., glaucoma). Therefore, further investigation of AD-specific impacts on particular retinal regions is required to improve the feasibility of using OCT-based biomarkers for early AD diagnosis.

### 2.2. Electrophysiological Changes of the Retina in Early-Stage Alzheimer’s Disease

Besides structural changes of the retina in AD, emerging evidence suggests that functional changes of the electrophysiological properties in specific retinal regions and visual impairment are associated with AD [42,43]. The noninvasive pattern electroretinogram (PERG) and pattern visual evoked potential (PVEP) tests reveal the bioelectrical functions of the retina and optic nerve [17]. In response to stimulation, the standard transient PERG recording wave includes three peaks: negative, positive, and negative polarity waves appear at approximately 35 ms (N35), 50 ms (P50), and 95 ms (N95), respectively [44]. The PERG response mirrors the electrical activity of the central retinal and ganglion cells and can contribute to auxiliary diagnoses for various anterior visual pathway diseases [44,45,46,47,48]. Furthermore, the amplitude and peak latency of PVEP are associated with the number of functional optic nerve fibers, which can be used as an objective assessment of visual pathway connectivity [49,50]. In PVEP testing, the recording wave also contains 3 peaks at 75 ms (N75), 100 ms (P100), and 135 ms (N135); P100 mirrors the functional bioelectricity in the optic nerve [18,51]. Moreover, retinocortical time—the latency difference between the PERG positive wave (i.e., P50) and the PVEP positive wave (i.e., P100)—provides more-exact information concerning the visual pathway transmission in the retina [17,50,52]. Several studies show that the PERG and PVEP parameters are altered in patients with AD at the early stage, which is consistent with the occurrence of RGC degeneration and optic nerve dysfunction [17,18,53,54,55,56]. For example, compared to healthy controls, patients with mild AD showed increased implicit time of the P50 wave and decreased amplitudes of both the P50 and N95 waves, suggesting that RGC and optic nerve dysfunction occur in early-stage AD [17]. Meanwhile, compared to healthy controls, patients with early-stage AD showed significantly longer P100-wave latency in the PVEP test and significantly longer retinocortical time, indicating dysfunctions of the optic nerve and neural conduction in the postretinal visual pathway [17]. These results suggest that key PERG and PVEP parameters are potential AD biomarkers.

### 2.3. Amyloid-Beta Deposition in the Retina in Alzheimer’s Disease

Aβ plaque deposition in the brain is a key feature of AD pathology and a potential biomarker for AD diagnosis [57]. Aβ is produced from APP (β-amyloid precursor protein) processing via β-secretase and γ-secretase cleavage [58]. During APP processing, the expression and activity of the β-secretase enzyme BACE1 (β-site APP-cleaving enzyme 1) is critical in Aβ production [58]. In APP/PS1 transgenic mice (a transgenic mouse model of AD), obvious BACE1 expression is detected in the GCL of the retina at 3 months, spreading to the IPL and OPL at 6 and 8 months, respectively; meanwhile, only weak BACE1 expression is observed in wild-type mice at 6 and 8 months [59]. Furthermore, enhanced BACE1 expression in various brain areas such as the entorhinal cortex, hippocampus, and prefrontal cortex is observed in APP/PS1 mice at 6 and 8 months, which is much later than when altered BACE1 expression appears in the retina (Table 1). This suggests that retinal BACE1 abnormalities are an early pathological feature and a potential biomarker of AD [59].

Aβ burden results in a series of AD-related pathological cascades including synaptic dysfunction, neuronal inflammation, oxidative stress, neuronal apoptosis, and damage to the mitochondria and endoplasmic reticulum, which ultimately lead to neuronal loss in the brain and cognitive impairment [61,70,71,72,73]. Moreover, Aβ induces neurotoxicity and impairs the functions of various cells such as neurons, astrocytes, microglia, and vascular endothelial cells [74]. Electron microscopy of RGCs in the Tg2576 AD mouse model reveals significantly altered mitochondrial complexity accompanied by mitochondrial swelling with large inter-mitochondrial spaces and fragmented cristae, which may be caused by Aβ accumulation [41]. Meanwhile, one recent study investigating the proteins and signaling pathways affected by Aβ administration in retinal photoreceptor cells found that Aβ causes ribosomal dysfunction, cytoskeletal protein dysregulation, and oxidative phosphorylation alterations [75]. Furthermore, Aβ might induce neurodegeneration concomitant with elevated immunoreactivity of MCP-1-, F4/80-, and TUNEL-positive cells in the retinal GCL, indicating increased inflammation and cell apoptosis [76].

Interestingly, several studies have documented Aβ accumulation in various retinal layers including the GCL, RNFL, OPL, ONL, INL, and IPL in cases of definite AD (Table 1) [64,65]. One study analyzed the retinal plaques in patients with AD on flat mounts and cross-sections using anti-Aβ antibodies (i.e., 12F4, 6E10, 4G8, and 11A5-B10) or compounds (i.e., curcumin and Congo red). The results showed all patients with AD had higher levels of Aβ deposition than controls [65]. Flat mounts analysis revealed that in AD retinas, Aβ is mainly distributed in the middle and far periphery of the superior quadrants, consistent with the observed neuronal loss and RNFL thinning of the superior retina [65]. Furthermore, staining of Aβ deposits in cross-sections from the superior retina shows that Aβ mainly accumulates in the innermost retinal layers (i.e., the GCL, INL, IPL, and ONL), especially in the GCL nearby or inside vascular walls [65]. Another study reported clusters of Aβ deposits in whole-mounted postmortem retinas of both patients with definite AD and those with suspected early-stage AD, whereas no Aβ plaques were observed in controls [60]. This suggests that retinal Aβ plaques can occur several years before the clinical signs of AD appear.

Consistent with findings from patients with AD, retinal plaques have also been reported in pre-symptomatic 3xTg-AD [61], Tg2576 [62,77], PSAPP [62], APP/PS1 [60,76,78,79], and 5xFAD mouse models of AD [62,80] and in the APP^NL-G-F^ knock-in mouse model [63]. For example, in APP/PS1 mice, both APP and Aβ deposition were detected in the retina including the RNFL, GCL, IPL, OPL, and INL (Table 1) [60,76,78,79]. More interestingly, in APP/PS1 mice, retinal Aβ plaques can be found as early as 2.5 months while brain plaques first appear at 5 months, indicating that retinal Aβ plaques emerge before brain plaques [60].

From a wholistic perspective, the presence of retinal Aβ plaques in AD remains controversial. While many studies report retinal Aβ plaques in animal models of AD or human patients, other studies report no Aβ plaques in the retinas of patients with definite AD [20,81,82,83], or APP/PS1 or Tg2576 mice [81]. This inconsistency may be due to differences in retinal regions, variable expression of retinal pathology among patients with AD, or differences in technologies or detection conditions such as Aβ immunofluorescent antibodies and chemical compounds [82,83].

PET imaging is a minimally invasive method that detects Aβ plaques and is therefore important for the clinical diagnosis of AD, although inaccessibility and cost hinder its population-scale application [84]. To detect Aβ oligomers, several researchers have attempted to exploit antibodies (e.g., 12F4, 6E10, 4G8, and 11A5-B10) and chemicals that bind to Aβ (e.g., curcumin, thioflavin, and Congo red). In particular, curcumin, a natural fluorescent compound with a high affinity for the β-pleated sheet structure of Aβ along with its oligomers, fibrils, and plaques, can pass through the blood–brain and blood–retina barriers after systemic administration [60,85,86]. In addition, clinical trials of curcumin have confirmed its low toxicity and safety, even at high dosages [87]. Retinal imaging technologies are used to visualize retinal Aβ plaques after curcumin administration in vivo. Curcumin staining is also used to examine postmortem retinas. Thus, curcumin specifically labels the retinal Aβ plaques, providing a noninvasive and feasible strategy for detecting early AD [60].

Prompted by a lack of methods to directly determine and quantify Aβ aggregates or fibrils, hyperspectral imaging (HSI) was developed to obtain spectral and spatial information about the retina by examining retinal Aβ without extrinsic fluorescent labeling (which binds Aβ), but rather through scanning within a certain wavelength range (i.e., visible to near-infrared) [84,88]. Thus, HSI spectral signatures can delineate the structural and biochemical variations of tissues, providing physiological, morphological, and histological diagnostic information for clinical applications [89]. Multiple studies using HSI have helped construct a database illustrating the spectral signature of Aβ aggregates [88,90]. Furthermore, the Aβ signature is associated with the order and kinetics of neuron-damaging Aβ aggregation, but not with monomeric or insoluble Aβ [90]. Upon using the DROP-D (Dimension Reduction by Orthogonal Projection for Discrimination) spectral analysis method to correct for inherent spectral variability, AD cases and controls exhibited different spectra according to the spectral data of Aβ in solution [84]. HSI analysis can identify Aβ-containing assemblies within the retina, specifically soluble Aβ aggregates in patients with AD, and thus generate the spectral signatures that discriminate patients with AD from healthy controls [84,88]. Interestingly, changes in the retinal HSI signature are much more dramatic in MCI cohorts than in AD cohorts with moderate cognitive impairment, suggesting that using HSI to detect retinal Aβ is a promising biomarker for early-stage AD [90]. Importantly, the retinal HSI signature is affected by structural alterations and biochemical variations such as hyperphosphorylated tau protein, iron accumulation, and inflammation; however, it is unaffected by other ophthalmological disorders including cataracts and glaucoma and is minimally affected upon aging [90,91]. Therefore, the development of HSI-based retinal biomarkers is a promising and sensitive method for large-scale preclinical AD risk screening.

### 2.4. Hyperphosphorylated Tau Aggregation

The pathological aggregation of hyperphosphorylated tau protein, which forms neurofibrillary tangles, is strongly correlated with cognitive decline in AD [92,93,94]. Hyperphosphorylated tau dissociates from microtubules, resulting in the destabilization of the microtubules and aberrations in neuronal functions [95]. Levels of hyperphosphorylated tau protein in the CSF and tau aggregation shown in brain PET imaging are considered biomarkers for clinical AD diagnosis [93,96].

Tau and its hyperphosphorylated forms have been detected in the retinas of patients with AD [3]. One study that assessed postmortem retinas in AD cases showed that total tau protein (detected by the HT7 antibody) is distributed in the retinal IPL and OPL, which are enriched with axonal connections [83]. Importantly, hyperphosphorylated tau with different phospho-epitopes (i.e., AT8, AT100, and AT270) is elevated in the OPL and IPL in patients with AD compared to that in controls [83]. Hyperphosphorylated tau protein immunoreactivity (i.e., AT8) is especially more obvious in the superior retina than the mid-retina and is distributed in a gradient increasing toward the periphery [83].

Consistent with these findings in humans, an accumulation of hyperphosphorylated tau aggregates is observed in AD mouse models [77,97,98,99]. For example, early retinal tau accumulation has been found in 3-month-old 3xTg-AD mice, preceding the occurrence of cognitive defects and tau aggregates in the brain [99]. Of note, retinal tau is distributed in the dendrites, soma, and intraretinal axons of RGCs, but is markedly decreased in the optic nerve axons in 3xTg-AD mice, suggesting that in early-stage AD, tau accumulation is lower in RGC axons within the optic nerve [99]. Retinal tau accumulation in early-stage AD can lead to neuronal impairment. Anterograde transport from RGCs to the superior colliculus in the brain is significantly higher in 3-month-old 3xTg-AD mice treated with tau-targeting siRNA, suggesting that the reduction of retinal tau partially restores RGC axonal transport [99]. Another study indicates that tau hyperphosphorylation (i.e., at Thr231, Thr205, or Ser396) is elevated in the retina in APP/PS1 transgenic mice [100]. Interestingly, this increased hyperphosphorylated tau in the retina of APP/PS1 transgenic mice is related to the upregulation of p35/p25 and activation of Cdk5 (cyclin-dependent kinase 5), which is mediated by calpain [100]. Furthermore, oligomeric tau is observed in the retina of tauopathy mice (i.e., P301L tau transgenic mice) and patients with AD or frontotemporal lobar dementia and is colocalized with Iba1-labeled microglia, GFAP-labeled astrocytes, and the pro-inflammatory cytokine HMGB1; these findings suggest that tau aggregates are associated with retinal inflammation [101]. Concordantly, neurofibrillary tangle levels are remarkably elevated in APP/PS1 mice compared to controls [100]. Nevertheless, the changes of neurofibrillary tangles in the retina remain controversial, as some studies report a lack of fibrillary tau, paired helical filaments, or neurofibrillary tangles in AD or tauopathies [81,83]. In addition, tau abnormality is also detected in glaucoma, which shares some characteristics with AD, such as optic nerve degeneration and visual pathway impairment [102].

In summary, hyperphosphorylated tau aggregates in the retina and is implicated in the pathogenesis of AD-related phenotypes, suggesting that retinal tau is a potential biomarker for early-stage AD. Schön et al. detected fibrillary tau aggregates by using scanning laser ophthalmoscopy after systemic administration of FSB ([E,E]-1-fluoro-2,5-bis [3-hydroxycarbonyl-4-hydroxy]styrylbenzene) to label the cells containing fibrillary tau and found that FSB-positive RGCs increased in the retina of P301S transgenic mice in vivo [81]. However, effective applications for detecting retinal tau in patients with AD are currently unavailable.

### 2.5. Blood Vessels and Retinal Microcirculation

Cerebrovascular disease might lead to cognitive impairment and dementia in elderly patients [103]. In fact, there are some overlapping symptoms between AD and cerebrovascular disease. Furthermore, many patients with AD also have cerebrovascular disease, vascular pathology, or moderate-to-severe cerebral amyloid angiopathy, as shown in the National Alzheimer’s Coordinating Centre Minimum Data Set [104]. Neuropathological studies show that large numbers of patients with vascular dementia have AD pathological characteristics [105], making it a challenge to distinguish between AD and mixed dementia in clinical settings. Interestingly, vascular impairments such as cerebral vascular infarction, arteriolar and venous stenosis, cerebral microbleeds, and capillary density reduction are key etiological factors involved in the development of early-stage AD [106]. Therefore, if vascular changes are established in AD cases, they can potentially be used as diagnostic criteria for AD [3].

The cerebral and retinal vasculatures have similar developmental origins, anatomical characteristics, and functional features, and retinal vascular abnormalities also occur in AD [107,108]. Accumulating evidence indicates that various retinal vascular parameters (RVPs) are related with AD-associated changes. Recent studies suggest that emerging retinal vascular alterations might mirror the cerebrovascular pathophysiological processes in AD [3,107,109]. For example, in a case–control study, the retinas of patients with mild-to-moderate probable AD (including those with mild or moderate dementia) exhibited obvious shrinkage of the venous blood column diameter and decreased venous blood flow rate compared to controls [23]. Furthermore, a recent study analyzed the retinal microvascular network of healthy subjects, patients with MCI, and patients with AD by quantitative analysis of the foveal avascular zone area and examination of the densities of the superficial retinal capillary plexuses (SRCPs) and deep retinal capillary plexuses (DRCPs) [110]. The results showed that the density of retinal microvasculature decreased in the DRCPs in all parafoveal and perifoveal quadrants in patients with AD; moreover, microvascular density was significantly reduced in most parafoveal quadrants (i.e., superior, temporal, and nasal) of the internal annular zone and in the superior external annular zone of the perifovea in the DRCPs in patients with MCI compared to healthy controls [110]. This suggests that retinal microvascular density in the DRCPs is a promising biomarker for early-stage AD. Another study reported that compared to controls, patients with AD have narrower venular calibers, reduced arteriolar and venular fractal dimensions, and increased tortuosity of arterioles and venules [107]. Consistently, the tortuosity of the retinal venules and arterioles is remarkably higher in Aβ-positive individuals than Aβ-negative individuals [111], corroborating the correlation between retinal vascular tortuosity and AD pathology. However, in one study, retinal imaging revealed decreased arteriolar tortuosity in patients with AD compared to controls [109]; this discrepancy might be due to other factors influencing the vascular tortuous process—for example, genetic differences, changes in vascular wall properties, or degenerative vascular disease [109]. Frost et al. examined RVPs in heathy subjects with high or low brain amyloid (the high brain amyloid group represents individuals in the preclinical stage of AD) and found that venule asymmetry factor and arteriole length diameter ratio were higher in the high brain amyloid group [112]. These findings indicate that RVPs changes are involved in the early stage of AD pathogenesis. Meanwhile, the study showed that a logistic model combining RVPs provides more accurate classification for the AD group than a logistic model combining age and *AopE*4 carrier status [112]. Thus, these RVPs (to a certain extent) are potential biomarkers for AD.

Most patients with AD exhibit comorbid cerebral amyloid angiopathy featuring Aβ in cerebral vessels [113], suggesting that cerebrovascular Aβ accumulation is associated with both AD and cerebral amyloid angiopathy pathology [114,115,116]. Pericytes and vascular cells control the blood flow of capillaries and maintain blood–brain barrier permeability, which is important for Aβ clearance in the brain [117,118]. The neural retina has a similar vasculature and blood barrier containing endothelial cells, pericytes, and astrocytes. Patients with AD, but not healthy controls, exhibit Aβ deposition within the vasculature and pericytes accompanied by pericyte loss [119]. In particular, PDGFRβ (platelet-derived growth factor receptor-β) in the vertical vessels of postmortem retinas is significantly lower in patients with MCI or AD than in cognitively normal controls and is correlated with increased in retinal vascular Aβ_40_ and Aβ_42_ in MCI and AD [119]. These findings imply that the blood–retina barrier mediates the retinal vascular disruption involved in AD and that retinal vascular parameters (e.g., vascular Aβ and PDGFRβ signaling) might be early signs of the occurrence of AD. Nevertheless, damage to the blood–retina barrier, which regulates retinal metabolism and vascular leakage, is a crucial factor in other retinal microvascular disorders such as AMD [120], diabetic macular edema [121], diabetic retinopathy [122], and uveitis [123]. Therefore, retinal vascular disruption may be nonspecific for AD detection.

Contrary to reduced density of deep vascular plexus (DVP) being reported in patients with MCI or AD, some studies found no significant difference in DVP between patients with AD and cognitively normal controls [110,124,125]. Many factors including type 2 diabetes mellitus, the detection equipment being used, and differing neurocognitive assessments may explain these contradictory results [124]. To overcome the variations caused by manual measurement and analysis, an automated modular machine learning pipeline that includes image quality selection, vessel map segmentation, and final classifier was recently developed to achieve AD classification based on the retinal vasculature changes [126]. In this way, the proposed pipeline could differentiate patients with AD from healthy controls efficiently and with high accuracy; meanwhile, the generated saliency map showed the small blood vessel was more important for classifying AD, which is consistent with previous studies showing the vessel features of AD progression [126,127,128].

Certain technologies have been used to examine vascular alterations in the retina in patients with AD. Retinal photography, a routine method for examining the retina, can reveal qualitative vascular signs including variation in the retinal vascular caliber, global geometrical patterns, and retinopathy [129]. A software that can quantitatively measure RVPs such as tortuosity and caliber provides a more effective evaluation of the vascular changes [130]. Of note, AD shares some common retinal changes with other diseases, including variations in vessel caliber (e.g., in AMD, stroke, diabetic retinopathy, and atherosclerosis), limiting the application of single retinal photographs for AD screening [13,131]. Nevertheless, advanced longitudinal parameters of retinal alterations based on retinal photography could improve the accuracy of identifying early-stage AD.

Furthermore, early microvascular abnormalities in AD might be identified using OCT–angiography followed by software-based quantification of the microvascular density of the retinal zone [132]. Moreover, various ophthalmological diseases can be assessed using Doppler OCT and laser Doppler blood flowmeter, which measure retinal blood flow [133]. One study showed that retinal venous blood flow assessed by laser Doppler blood flowmeter is significantly lower in patients with AD or MCI than in healthy controls [134]. Consistent with these findings, a Doppler OCT study showed that retinal arterial and venous blood flow are significantly reduced in patients with AD or MCI compared to controls [135]. Given that the tortuosity of the retinal arterioles and venules as well as the diameter and textures of vessels are altered in AD, combining these vascular features and spatial–spectral texture data from different retinal regions by hyperspectral retinal imaging can facilitate the classification accuracy between Aβ-positive and Aβ-negative groups [111].

### 2.6. Apoptosis in the Retina in Alzheimer’s Disease

Besides the various retinal changes reported in AD, corresponding molecular pathways are implicated in AD pathogenesis. Given that the Aβ accumulation is observed in several retinal layers in AD, Aβ may be associated with RGC death in AD, AMD, and glaucoma [136]. Aβ peptides exhibit toxicity toward neurons and glia and can result in apoptosis associated with neuronal loss in AD [137,138]. Accordingly, cell death markers can be used to trace apoptotic cells within the retina in vivo. New imaging techniques involving labeling with ANX776 (annexin 5 labeled with fluorochrome DY-776), Zn-DPA (bis[zinc{II}]-dipicolylamine), and other fluorescent dyes can monitor apoptotic cells [139,140,141]. For example, Zn-DPA can bind to the phosphatidylserine that is exposed to cell membranes during early-stage apoptosis and be detected by fluorescence imaging after intravitreal injection [141]. Another study showed that retinal cell apoptosis could be monitored in mice and rats by fluorescence imaging using Zn-DPA conjugated with Texas red (PSVue-550) administered noninvasively by eye drops [140]. ANX776 has been verified as safe with no adverse effects in humans after intravenous injection and has a short half-life of 10–36 min [139]. Intravenous injection of ANX776 combined with DARC (detection of apoptosing retinal cells) imaging can allow to visualization of retinal cell apoptosis (as spots labeled by ANX776) in real time in vivo for glaucoma and other neurodegenerative disorders such as AD and Parkinson’s disease [139]. These findings suggest that this technology can help monitor early-stage retinal apoptosis in vivo.

## 3. Alzheimer’s Disease and Other Retinal Diseases

Aberrant retinal changes in AD are potential novel biomarkers for early-stage AD. Of note, several ophthalmological disorders including AMD and glaucoma may partially overlap with AD owing to their shared characteristics such as neuron degeneration, Aβ deposition, and microvascular abnormalities [142,143].

### 3.1. Retinal Dysfunction in Age-Related Macular Degeneration

AMD is a common retinal degenerative disease in the elderly population [144]. Early-stage AMD features the emergence of drusen and RPE degeneration, while late-stage AMD features geographic atrophy and choroidal neovascularization [144]. Some studies suggest that AMD and AD might be comorbid by showing that patients with AMD have an increased risk of AD [145,146,147], although this correlation becomes not significant when considering other factors such as age, presence of the apolipoprotein E (*ApoE*) allele, smoking, and atherosclerosis [145,148]. Evidence that Aβ deposition is involved in chronic inflammation, oxidative stress, and neuronal damage in both AD and AMD, suggests that the pathological similarities between these diseases, to some extent, may reduce the specificity of potential biomarkers for AD diagnosis [149].

Investigations of the pathological features of AD and AMD reveals several retina-related parameters that can differentiate these two diseases. For example, retinal Aβ deposits appear in both AMD and AD. In patients with AD, Aβ deposition is observed in many retinal layers such as the GCL, RNFL, OPL, ONL, INL, and IPL. In contrast, in AMD, Aβ deposits are associated with drusen, which are formed by various sediments and exist in the retinal pigment epithelium and Bruch’s membrane [150,151]. In addition, retinal degeneration in AMD occurs mainly in the macular, RPE, and photoreceptor layers [144], whereas retinal degeneration in AD predominantly occurs in the RNFL and GCL [152]. Moreover, the vascular changes in AMD, which feature choroidal neovascularization (detected by fluorescein angiography), are not reported in AD [153,154]. Furthermore, the *ApoE* gene is associated with both AMD and AD [155,156]. There are three known isoforms of *ApoE*: *ApoE2*, *ApoE3*, and *ApoE4* [155]. Interestingly, the *ApoE4* isoform is a genetic risk factor for AD and might be associated with cognitive function; conversely, *ApoE2* serves as a protective element against AD [157,158]. However, *ApoE2* is considered a risk gene that promotes AMD progression, whereas *ApoE4* does the opposite [156]. These different features could be used to develop new strategies to distinguish AD and AMD and develop disease-specific biomarkers.

### 3.2. Retinal Dysfunction in Glaucoma

Glaucoma is another ophthalmological disorder that features RGC degeneration and optic nerve damage [159]. Glaucoma is reported to significantly increase the risk of AD [146,160], and the occurrence of glaucoma is significantly elevated among patients with AD [161]. Both of these diseases involve progressive degeneration and loss of RGCs [162]. Similar to the Aβ and tau aggregation observed in the AD retina, Aβ and hyperphosphorylated tau along with RGC degeneration are also reportedly involved in glaucoma pathology [102,136,163]. Aβ was observed in the optic nerve and RGC layer in the glaucoma DBA/2J mouse model and might contribute to RGC loss [164]. Consistently, several studies indicate that RGC death is related to Aβ toxicity in glaucoma [136,165]. In human glaucoma, abnormal tau (AT8) is localized in the INL [102], and in the rat glaucoma model, retinal tau (K9JA) accumulates dramatically in the IPL and is modestly increased in the GCL [166], while tau accumulates in the OPL, GCL, IPL, and RNFL of the retina in AD. AD and glaucoma share other similarities such as GCL thickness and blood–retinal barrier breakdown [163], making these retinal biomarkers nonspecific for the early identification of AD.

However, the vessel density of DVP was decreased in patients with AD, whereas the vessel density of the superficial vascular plexus and radial peripapillary capillary were reduced in patients with primary open-angle glaucoma [167]. Importantly, intraocular pressure (IOP) is considered a significant factor in the development of glaucoma [168], but the incidence of ocular hypertension was absent in patients with AD [161], suggesting that IOP is not associated with AD pathology and may be a pivotal factor in differentiating AD and glaucoma.

### 3.3. Strategies to Distinguish Alzheimer’s Disease from Age-Related Macular Degeneration and Glaucoma

Given that AD shares similarities with AMD and glaucoma (Table 2), a single retinal biomarker may be insufficient for detecting AD. Nevertheless, it is possible to distinguish AD from other ophthalmological diseases through combining multidimensional biomarkers or examining other retinal changes to exclude AMD and glaucoma. For example, multimodal imaging—including color photography, spectral-domain OCT, and fluorescein angiography—is used to detect specific symptoms of AMD such as drusen, pigment abnormalities, geographic atrophy, and neovascularization [144]. In contrast, these symptoms do not appear in AD. In addition, the differences in the optic nerve head between AD and glaucoma measured by confocal scanning laser tomography can successfully differentiate glaucoma from AD [169]. Meanwhile, as high IOP is an important factor in glaucoma progression, IOP examination may be used to differentiate AD and glaucoma. A recent study shows that combining quantitative assessment of inner retinal layer and outer retinal layer thickness with DVP density may be a helpful approach for differentiating AD and primary open-angle glaucoma [167]. Furthermore, combining other AD-associated biomarkers, including CSF Aβ, tau, and amyloid PET, with structural MRI may be an alternative way to distinguish AD from other disorders, including ophthalmological diseases [170].

## 4. Future Directions and Challenges

**Figure 1:**
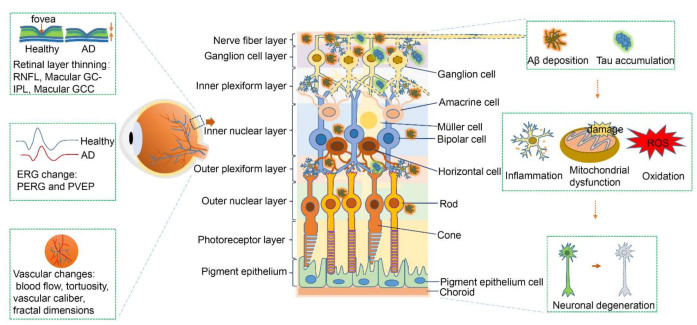
Schematic summary of retinal abnormalities in Alzheimer’s disease. In Alzheimer’s disease (AD) pathogenesis, amyloid-beta (Aβ) deposition and tau aggregation induce pathological cascades including neuronal inflammation as well as mitochondrial dysfunction and oxidation, which ultimately result in neuronal loss and degeneration. Meanwhile, in AD, the retina manifests electroretinographic changes, retinal thinning, and vascular changes. ERG, electroretinogram; GCC, ganglion cell complex; GC-IPL, ganglion cell–inner plexiform layer; PERG, pattern electroretinogram; PVEP, pattern visual evoked potential; RNFL, retinal nerve fiber layer; ROS, reactive oxygen species.

Given that the retina is a “window” into the brain and a good means of further investigating AD pathophysiology, examining retinal changes could be important for detecting early-stage AD. In this review, we highlighted several retinal abnormalities in AD—such as structural changes, alteration of electrophysiological properties, vascular variations, and Aβ and tau accumulation—that ultimately lead to neuronal loss (Figure 1). Thus, using potential retinal biomarkers to detect early AD might be beneficial for patients with AD by enabling early intervention and consequently retarding disease progression (Table 3 and Table 4). Although some studies propose monitoring retinal changes as a promising option for large-scale screening of the AD patient population, establishing specific and accurate retinal biomarkers for detecting early-stage AD is challenging. Some retinal biomarkers may be inadequate and nonspecific for detecting AD, for example, because of overlapping changes between AD and other diseases such as AMD, glaucoma, vascular dementia, and Parkinson’s disease. Meanwhile, other factors influence retinal alterations, such as genetics, age, and other comorbidities. Variation of the pathological features across patients with AD also increases the challenge of differentiating early-stage AD individuals from healthy individuals (Table 5).

Future studies using standardized protocols across different cohorts, in combination with patient stratification by precision medicine approaches, will help solve these discrepancies. To discover the specific retinal changes in AD pathology, future research can be directed to analyzing the retinal abnormalities in a larger population of patients through standardized classification—adjusting for age, race, genetics, and excluding other neurological or ophthalmological diseases—and then use of standard methodologies for further analysis. Moreover, although Aβ and tau are reportedly present in some layers of the retina, whether they are specifically localized in different cell types remains unclear. Hence, further studies are required to clarify the molecular mechanisms underlying retinal changes in AD as well as to improve the accuracy and universality of retinal biomarkers for AD.

## Figures and Tables

**Table 1:** Sequential occurrence of amyloid-beta deposition and BACE1 appearance in the retina and the brain in Alzheimer’s disease.

Molecular Changes	Retina	Brain
Aβ deposition	In the retina at 2.5 months in APP/PS1 mice [60] In the GCL of the retina at 5–10 post-natal weeks in 3xTg-AD mice [61] Senile plaque appeared in the retina at 2 months in 5xFAD mice and at 6 months in PSAPP mice [62] Soluble Aβ_42_ appeared at 3 months in APP^NL-G-F^ knock-in mice [63] In the GCL, RNFL, OPL, ONL, INL, and IPL of the retina in patients with AD [64,65] and in the whole-mounted retinas of patients with definite AD or suspected early-stage AD [60]	Appears at 5 months in APP/PS1 mice [60] Distributes in the neocortex, entorhinal region, hippocampus, diencephalic, brain stem, and cerebellar regions of the brain in patients with AD [66]
BACE1 expression	In the GCL of the retina at 3 months and then spreads to the IPL and OPL at 6 and 8 months, respectively, in APP/PS1 mice [59]	In the entorhinal cortex, hippocampus, and prefrontal cortex at 6 and 8 months in APP/PS1 mice [59] Increased expression of BACE1 in the temporal cortex and hippocampus in the AD brain [67,68] High activity of BACE1 in the brain in patients with sporadic AD [67] High activity of BACE1 in the cerebrospinal fluid in patients with MCI or AD [69]

Aβ, amyloid-beta; AD, Alzheimer’s disease; BACE1, β-site APP-cleaving enzyme 1; GCL, ganglion cell layer; INL, inner nuclear layer; IPL, inner plexiform layer; MCI, mild cognitive impairment; ONL, outer nuclear layer; OPL, outer plexiform layer; RNFL, retinal nerve fiber layer.

**Table 2:** Comparison of retinal changes in Alzheimer’s disease, age-related macular degeneration, and glaucoma.

Symptom	AD	AMD	Glaucoma
Retinal thickness	Thinning in the RNFL, GC-IPL, and GCC of the retina [29,31,171,172]	Thinning in the macular region and photoreceptor layers [173]	Thinning in the GCL and RNFL of the retina [163]
Electrophysiology	PERG and PVEP changes [17,18]	Full-field ERG and multifocal ERG changes [174]	PERG and PVEP changes [175]
Aβ deposition	In the inner retina [64,65,143]	Along with drusen [150,151]	In the optic nerve and RGC layer of the retina [164]
Tau accumulation	In the OPL, IPL, GCL, and RNFL of the retina [3,83]	N	In the INL, IPL, and GCL of the retina [102,166]
Apoptosis	RGC apoptosis [176]	Apoptosis of the RPE, photoreceptors, and INL cells [177]	RGC apoptosis [178]
Ocular hypertension	Ocular hypertension was absent in patients with AD [161]	N	Elevated IOP in high-tension glaucoma and normal IOP in normal-tension glaucoma [168,179]
Hyperpigmentation	N	Hyperpigmentation is present in AMD patients [180]	N
Geographic atrophy	N	Geographic atrophy surround and spare the central macula [144]	N
Choroidal neovascularization	N	Choroidal neovascularization is present in neovascular AMD [181]	N

Aβ, amyloid-beta; AD, Alzheimer’s disease; AMD, age-related macular degeneration; ERG, electroretinogram; GCC, ganglion cell complex; GC-IPL, ganglion cell–inner plexiform layer; GCL, ganglion cell layer; INL, inner nuclear layer; IOP, intraocular pressure; IPL, inner plexiform layer; MCI, mild cognitive impairment; N, Not found; OPL, outer plexiform layer; PERG, pattern electroretinogram; PVEP, pattern visual evoked potential; RGC, retinal ganglion cell; RNFL, retinal nerve fiber layer; RPE, retinal pigment epithelium.

**Table 3:** Description of potential techniques for monitoring Alzheimer’s disease.

Techniques	Description	Main Findings
Spectral-domain optical coherence tomography	A noninvasive method that provides high-resolution images of retinal morphological structures and volumetric parameters [130]	Reduction in RNFL thickness [29,31,171,172] Decrease in macular thickness and volume [31] Reduction in macular GC-IPL thickness [29,31] Reduction in macular GCC thickness [31,32]
Optical coherence tomography–angiography	A noninvasive method that can accurately visualize the vascular system of different retinal layers at a 3-dimensional level [110]	Decrease in the microvascular density of deep retinal capillary plexuses [110]
Doppler optical coherence tomography and laser Doppler blood flowmeter	Noninvasive methods that can visualize and quantify blood flow [133]	Reduction in venous blood flow [134] Decrease in retinal arterial blood flow [135]
Retinal photography (ocular fundus)	A noninvasive method that provides retinal images and vascular signs [130]	Narrowing of venular calibers [107] Reduction in arteriolar and venular fractal dimensions [107,109] Increase in tortuosity of arterioles and venules [107] Detection of retinal Aβ plaques after curcumin administration [60]
Pattern electroretinogram and pattern visual evoked potential	Noninvasive methods that reflect the bioelectrical functions of the retina and optic nerve related to visual pathway transmission [17]	Increase in implicit time of P50 [17,18] Amplitude reduction in both P50 and N95 [17,18] Increase in P100-wave latency [17,18] Increase in retinocortical time [17]
Hyperspectral imaging	A noninvasive method that detects retinal Aβ without extrinsic fluorescence labeling by scanning within a specific wavelength range [88]	Detection of retinal Aβ signature [84,90]
Detection of apoptosing retinal cells	A method that allows real-time observation of retinal apoptotic cells in vivo [139]	RGC apoptosis in 3×Tg-AD mice (a mouse model of AD) [176]

Aβ, amyloid-beta; AD, Alzheimer’s disease; GCC, ganglion cell complex; GC-IPL, ganglion cell–inner plexiform layer; RGC, retinal ganglion cell; RNFL, retinal nerve fiber layer.

**Table 4:** Potential retinal biomarkers for Alzheimer’s disease.

Pathology	Detection Methods	Expected Outcomes
AD pathology		
Amyloid deposition	Retinal imaging Hyperspectral imaging	Detects retinal Aβ plaques after curcumin administration [60] Detects retinal Aβ signature [84,90]
BACE1 expression	Currently unavailable in patients with AD	No information
Tau aggregation	Currently unavailable in patients with AD	No information
Non-AD pathology		
Structural changes		
RNFL thickness	Optical coherence tomography	Decrease in RNFL thickness [29,31,171,172]
GC-IPL thickness		Decrease in macular GC-IPL thickness [29,31]
Macular GCC thickness		Decrease in macular GCC thickness [31,32]
Electrophysiological changes		
PERG parameters	PERG examination	Increase in implicit time of P50 [17,18] Amplitude reduction in both P50 and N95 [17,18]
PVEP parameters	PVEP examination	Increase in P100-wave latency [17,18]
Vascular changes		
RVPs	Retinal photography Doppler optical coherence tomography and laser Doppler blood flowmeter	Narrowing of venular calibers [107] Reduction in arteriolar and venular fractal dimensions [107,109] Increase in tortuosity of arterioles and venules [107]
Apoptosis	Detection of apoptosing retinal cells in vivo Currently unavailable in patients with AD	Detected RGC apoptosis in 3×Tg-AD mice (a mouse model of AD) [176]

Aβ, amyloid-beta; AD, Alzheimer’s disease; BACE1, β-site APP-cleaving enzyme 1; GCC, ganglion cell complex; GC-IPL, ganglion cell–inner plexiform layer; PERG, pattern electroretinogram; PVEP, pattern visual evoked potential; RGC, retinal ganglion cell; RNFL, retinal nerve fiber layer; RVPs, retinal vascular parameters.

**Table 5:** Comparison of different studies on the retinal changes in Alzheimer’s disease.

Retinal Indicators	Results	Contradictory Findings
Retinal thickness	Reduction in RNFL, GC-IPL, and macular GCC thickness [29,31,171,172]	No differences in macular retinal layer thickness and pRNFL thickness [33,34]
Aβ plaques	Retinal Aβ plaques in patients with AD [64,65,143]	No Aβ plaques in the retina in patients with AD [20,81,82,83], or APP/PS1 or Tg2576 mice [81]
BACE1 expression	Detected in the retina of APP/PS1 mice [59]	N
Tau aggregation	Hyperphosphorylated tau and total tau found in the retina in patients with AD [3,83]	No fibrillary tau, paired helical filaments, or neurofibrillary tangles in AD [81,83]
PERG and PVEP	Changes in PERG and PVEP parameters [17,18]	No changes in PERG and PVEP parameters [182]
Retinal vasculature changes	Decrease in the retinal microvascular density of the DRCPs [110] Narrowing of venular calibers [107] Reduction in arteriolar and venular fractal dimensions [107,109] Increase in tortuosity of arterioles and venules [107] Decrease in venous blood flow and retinal arterial blood flow [134]	Decrease in arteriolar tortuosity [109] No significant change in the density of DVP [124,125]
RGC apoptosis	RGC apoptosis in 3×Tg-AD mice (a mouse model of AD) [176]	N

Aβ, amyloid-beta; AD, Alzheimer’s disease; BACE1, beta-site amyloid precursor protein cleaving enzyme 1; DRCPs, deep retinal capillary plexuses; GCC, ganglion cell complex; GC-IPL, ganglion cell–inner plexiform layer; N, Not found; PERG, pattern electroretinogram; pRNFL, peripapillary RNFL; PVEP, pattern visual evoked potential; RGC, retinal ganglion cell; RNFL, retinal nerve fiber layer; DVP, deep vascular plexus.
